# Histopathological spectrum of cutaneous neoplasms - A retrospective study

**DOI:** 10.6026/973206300212249

**Published:** 2025-07-31

**Authors:** Garima Anandani, Parth Goswami, Payal Bhatt, Vaishali Bhankhodia, Yashdeep Singh Pathania

**Affiliations:** 1 Department of Pathology, AIIMS Rajkot, Gujarat - 360110, India; 2 Department of Dermatology, AIIMS Rajkot, Gujarat - 360110, India

**Keywords:** Skin neoplasms, benign, premalignant, malignant, histopathology, clinical correlation

## Abstract

Cutaneous neoplasms exhibit a wide histopathological spectrum, often posing diagnostic challenges due to overlapping clinical features. This retrospective study analyzed 66 cases diagnosed between January 2022 and December 2024 at a tertiary care center in Gujarat. Benign tumors were most common (69.7%), with keratinocytic tumors being the predominant type (40.9%). The face was the most frequently affected site (34.9%), and nodular lesions were the typical presentation (63.6%). Histopathology remains essential for accurate diagnosis and timely treatment of skin tumors.

## Background:

Skincancer is technically not very prevalent as a malignancy in the world, but the incidence of these skin tumors has been increasing tremendously over the past decades [1]. The lesions that are classified under these neoplasms constitute a very broad category of lesions that is very heterogeneous in its histological and clinical composition and as such pose a significant diagnostic dilemma [2]. Cutaneous tumors are broadly divided into neoplasms of the keratinocytic origin, which involve, among others, basal cell carcinoma, squamous cell carcinoma, melanocytic tumors that include their nevi and melanoma, and adnexal tumor which includes the sweat glands, sebaceous glands, and hair follicle. Also, mesenchymal subcutaneous and skin tumors make a large share of the cutaneous neoplasm spectrum, the examples being dermatofibromas, lipomas, and tumors of a vascular aspect [4]. A more complicated type under this category consists of primary cutaneous epithelioid soft tissue tumors arising with those tumors have divergent lines of differentiation. These are tumors of melanocytic, peripheral nerve sheath, angiomatous, fibrohistiocytic, myoid, and myoepithelial origin that carry different considerations in terms of diagnostic and treatment settings [5]. Their morphology is overlapping and the behavior of the lesions is variable- with some of them showing an indolent benign and in some cases being aggressive malignancies- hence the histopathology should be carefully determined to get the correct diagnosis [6]. Due to the wide spectrum of biological behavior of these neoplasms and the possibility of malignant transformation of premalignant conditions it is critical to obtain accurate histological diagnosis that allows effective clinical decision-making and planning individual treatment [7]. Therefore, it is of interest to outline the histopathological spectrum of cutaneous neoplasms in a tertiary care center to enhance diagnostic accuracy in this regard and suggest the relevant management plans.

## Materials and Methods:

The present retrospective cross-sectional record-based study was conducted in Department of Pathology of a tertiary care center of Gujarat. Patients of any age or sex, who were clinically suspected to have a cutaneous neoplastic pathology and whose biopsy or excision was sent and diagnosed to be a neoplastic lesion, were included. These encompassed all the neoplastic lesions of skin including epidermal, dermal, subcutis or soft tissue lesion comprising of benign, premalignant and malignant entities. Cases who were suspected to have cutaneous neoplastic pathology but who were histopathologically diagnosed to be reactive or non-neoplastic entity were excluded. We compiled the retrospective record-based data of these 66 included patients who visited dermatology outpatient department at our hospital for consultation from January 2022 to December 2024. The histopathology slides including Haematoxylin and Eosin (H & E) as well as other special stains if available were retrieved for all the included cases. All these slides were re-examined for validation and confirmation of the diagnosis in hospital information system. Statistical analysis was done by Statistical Package for Social Sciences (SPSS) version 21.0. Continuous variables were presented as mean ± standard deviation (SD) and median. Categorical variables were presented as number and percentage (%).

## Results:

The spectrum of cutaneous neoplasms was categorized into eight distinct groups based on their cellular origin. These categories encompass keratinocytic, melanocytic, adnexal, vascular, fibrohistiocytic and myoid, neural and lymphoid lesions. The age of the patients were from ten to ninety years, with the highest frequency observed in the 31-40 years age group, which accounted for 14 cases (21.2%) followed by 41-50 years comprising of 13 cases (19.7%). The mean age of the patients was 47.60 ± 18.04 years. Amongst all the included cases, 57.6% were males and 42.4% were females. The most common age group for keratinocytic lesions was 41-50 years predominantly benign in nature, followed by 61-70 years with higher frequency of pre malignant and malignant lesions. Benign lesions were commoner in younger age groups compared to malignant lesions in older age groups. Adnexal, vascular, fibrohistiocytic and myoid lesions were common in 31-40 years age group, neural in 41- 50 years, lymphoid 51-60 years while cases showing melanocytic lesions had a variable age presentation. Males were more commonly affected in keratinocytic, adnexal, neural and myoid cutaneous lesions while females in melanocytic, fibrohistiocytic and lymphoid (Figure 1 - see PDF). However, there was 1:1 male: female ratio in vascular cutaneous tumors (Table 1 - see PDF). The most common site of presentation was face (34.9%) followed by upper limb (22.7%), lower limb (16.7%), scalp (10.6%), back (6%), neck (3%), genitals (3%), chest (1.55%) and abdomen (1.55%) (Table 2 - see PDF).

Keratinocytic tumors had a varied site of presentation of which face, upper limbs and lower limbs were most commonly affected. Adnexal tumors were common on face and scalp; melanocytic and myoid tumors on face; vascular tumors on scalp and lower limbs; fibrohistiocytic tumors in upper limbs; while lymphoid and neural tumors presented on back. Basal cell carcinoma (BCC) was commonly observed on the cheek and nose, whereas squamous cell carcinoma (SCC) was frequently found on the ear pinna and sebaceous carcinoma on the eyelids. Based on the duration of persistence of lesion, the cases were categorized as acute if the duration was ≤ 6 months and chronic if > 6 months. Chronic presentation was more common than the acute presentation in our cases of cutaneous neoplasms. Acute clinical presentation was usually seen in seborrheic keratosis, Pseudoepitheliomatous hyperplasia (PEH), BCC, SCC, hemangioma, cutaneous horn and mycosis fungoides (MF). Most common presentation was nodular swelling or growth (63.6%) (Table 3 - see PDF). Three cases with nodular presentation had hyperpigmented nodule, of which two turned out to be seborrhoeic keratosis and one benign cutaneous horn. One case of Wilson's disease acutely presented with plaque and turned out to be PEH. Two cases with presentation as a plaque had papules also along with plaque. One was Acrokeratosis Verruciformis of Hopf (AKV) and another was a clinically suspected case of lichen planus which turned out to be verruca vulgaris on histopathology. One case had a verrucous plaque which turned out to be nevus sebaceous of Jadassohn. Other three cases of plaque turned out to be pigmented BCC, nevus sebaceous and Pseudoepitheliomatous Keratotic and Micaceous Balanitis (PKMB). Out of seven cases with ulcer, three were pigmented BCC, one SCC, one verruca plantaris, one PEH and one MF. Two cases had a history of plaque before which converted to ulcer formation. One was a case of BCC and another MF. Out of seven cases of papules, three were hyperpigmented and two were hypopigmented. Hyperpigmented papules were seen in two cases of epidermal nevi and one congenital melanocytic nevus. Hypopigmented papules were seen in two cases of trichoepithelioma. One case of fibrokeratoma and one case of angiofibroma also presented with papules. There were three cases with warty growth of which two were condyloma and one actinic keratosis. Histopathological examination classified these cases into eight unique groups according to their cellular origin. Within each group, they were further divided based on the presence or absence of malignancy, specifically categorized as benign, premalignant, or malignant. A total of 69.7% of the cases were classified as benign, 9.1% as pre-malignant and 21.2% as malignant (Figure 2 - see PDF). Benign tumors like chondroid syringoma, trichoepithelioma, pilomatricoma, eccrine spiradenoma, eccrine hidrocystoma, lobular capillary hemangioma (LCH) (Figure 3 - see PDF), hemangiomas including capillary and cavernous types, angiofibroma, glomus tumor, acrochordon (benign fibroepithelial polyp), benign cutaneous horn, verruca vulgaris, verruca plantaris, myrmecia wart (Figure 4 - see PDF), seborrheic keratosis, AKV, PEH, PKMB, epidermal nevus, congenital melanocytic nevus, dermatofibroma, benign fibrous histiocytoma (BFH), juvenile xanthogranuloma (JXG), fibroblastic rheumatism, cutaneus leiomyoma, neuroma, neurofibroma (NF) and schwannoma were identified. Premalignant lesions comprised of condyloma, actinic keratosis, nevus sebaceous / nevus sebaceous of Jadassohn and proliferating pilar tumor. Malignant lesions included BCC, SCC, malignant melanoma, sebaceous carcinoma and MF. LCH represented the most frequently occurring benign tumor, while nevus sebaceous and condyloma were identified as the predominant premalignant lesions [3, 4]. BCC emerged as the most prevalent malignant lesion among the cases examined. Based on the cell of origin, keratinocytic tumors were most common (40.9%), followed by vascular tumors (18.2%) and adnexal (13.6%). The distribution of all the cases according to the site, clinical presentation and histopathology is depicted in Table 4 (see PDF).

## Discussion:

The skin consists of various cell types that operate in a mutually dependent and collaborative manner [5]. The epidermis primarily consists of keratinocytes, melanocytes, Langerhans cells and Merkel cells. Epidermal appendages, which extend from the epidermis into the dermis, include specialized cells such as follicular epithelial cells, sebaceous cells and the cells of eccrine and apocrine glands. Additionally, lesions in the dermis and the underlying subcutaneous and soft tissues may manifest clinically as skin tumors. Different skin tumour consists of different cells [6]. In routine clinic patient can have variety of benign, pre malignant or malignant tumours. Due to the heightened ultraviolet radiation caused by the depletion of the ozone layer, it is anticipated that the incidence rates of skin malignancies will rise in the future, unless changes in human behavior aimed at reducing sun exposure can mitigate these expected increases [7]. The precise identification of skin lesions is essential to prevent the oversight of malignancies and to ensure that they are addressed promptly, thereby reducing the risks of morbidity and mortality [8]. Skin biopsy serves as a crucial technique that aids clinicians in establishing a definitive diagnosis and directing patient management. In this research, benign tumors were found to be more prevalent than malignant tumors, accounting for 69.7%. This finding is consistent with previous studies conducted by Thapa *et al.* (57.34%), Shrivastava *et al.* (63.8%), Narhire *et al.* (69.4%) and Kaur *et al.* (67.27%) [1, 9, 10-11]. In contrast to the findings of this study, several researches conducted by Gundalli, Nandyal and Samanta have reported a higher prevalence of malignant tumors. This discrepancy may be attributed to an increased number of referrals to higher-level medical centers, as well as variations in geographical factors [12, 13-14]. Keratinocytic tumors were the most prevalent, accounting for 40.9% of cases, followed by vascular tumors at 18.2% and adnexal tumors at 13.6%. In contrast, the study conducted by Thapa *et al.* reported that keratinocytic tumors comprised the majority at 66.9%, with adnexal tumors at 19.0% and melanocytic tumors at 14.0%. This finding aligns with the results from Uplaonkar *et al.* where keratinocytic tumors represented 41.7%, appendageal tumors 38.9% and melanocytic tumors 19.4% [1, 15]. In the research conducted by Pappala *et al.* keratinocytic tumors accounted for 60.52%, melanocytic tumors represented 23.3% and appendageal tumors comprised 16.3% [16]. Similar to our study Goel *et al.* also found keratinocytic tumor as the most prevalent type of tumor [17].

Whereas in the research conducted by Gundalli *et al.* and Narhire *et al.* the most frequently observed tumor was the benign skin adnexal tumor, accounting for 54.7% [10, 12]. Several other studies identified benign melanocytic tumors as the most prevalent type of skin tumors [9, 14]. Research conducted by Kaur *et al.* indicated that cutaneous neoplasms are more prevalent in males compared to females, aligning with the findings of the current study [11, 17]. In contrast, few other studies indicated a predominance of females [1, 9 and 18]. In the current research, cutaneous neoplasms were identified in individuals aged between 10 and 90 years, with the highest prevalence noted during the fourth and fifth decades of life. Keratinocytic lesions were predominantly observed in the fifth decade, primarily exhibiting benign characteristics, while the seventh decade showed an increased occurrence of pre-malignant and malignant lesions. Adnexal, vascular, fibrohistiocytic and myoid lesions were frequently found in the fourth decade, neural lesions in the fifth decade and lymphoid lesions in the sixth decade. Cases involving melanocytic lesions displayed a diverse age distribution. Whereas in other studies, the majority of cutaneous tumors were reported in the third and sixth decades of life, with benign neoplasms being more prevalent among the younger population [11, 13, 18 and 19]. In the study done by Thapa *et al.* it was observed that most cutaneous tumors occurred in individuals during their sixth decade of life, in contrast to the study done by Sherpa and KC which identified the third decade as the most prevalent age group for these tumors [1, 19]. In the current study, the head and neck region was identified as the most frequently affected area, accounting for 48.5% of cases, with the face being the most commonly involved subregion. This was followed by the extremities, which represented 39.4% of the cases, consistent with findings from several other studies [1, 9, 10, 17 and 20]. It can conclude that sun exposed area have high chance of development of skin tumours. In our research, LCH emerged as the most prevalent benign cutaneous neoplasm, while acrochordon was identified by Thapa *et al.* squamous papilloma by Pappala *et al.* and verrucas by Shrivastava *et al.* Kaur *et al.* [1, 9, 11, 16 and 17]. Trichoepithelioma emerged as the predominant skin adnexal tumor in our research, differing from the findings of Gundalli *et al.* Thapa *et al.* Shrivastava *et al.* and Sherpa and KC, where pilomatricoma was more prevalent [1, 9, 12 and 19]. However in the study done by Gowda *et al.* syringoma represented the most prevalent type of cutaneous appendageal tumor [21]. BCC emerged as the predominant cutaneous malignant neoplasm in this study, aligning with the findings of Nair *et al.* while contrasting with the highest prevalence of SCC reported by Deprez *et al.* LeBoit *et al.* and Thapa *et al.* [1, 22, 23- 24]. Clinically, there exists a significant dilemma, as provisional diagnoses may differ markedly from the definitive histopathological findings, particularly due to the multitude of entities that can present similarly, especially among benign lesions. The current study illustrates this point, where a case initially suspected to be an epidermal cyst or mucocele was ultimately identified as a chondroid syringoma. Additionally, a lesion that raised suspicion for papilloma or a mucous retention cyst was confirmed to be a hemangioma. One case initially thought to be acrochordon was diagnosed to be a neuroma, while a lesion suspected to be either a cylindroma or dermatofibrosarcoma protuberans (DFSP) was found to be a neurofibroma. A patient presenting with a differential diagnosis of trichofolliculoma, trichoblastoma and sebaceoma was ultimately diagnosed with a benign fibrous histiocytoma (BFH). Furthermore, a case clinically suspected to be a lipoma or hematoma was confirmed as an eccrine spiradenoma and another case initially thought to be a sebaceous cyst was diagnosed as an eccrine hidrocystoma. Lastly, a case of verrucous vulgaris was clinically misidentified as lichen planus. (Figures 5 to 8 - see PDF) present photomicrographs of several cutaneous skin tumors, with details provided in the legends.

## Conclusion:

Diagnosing skin tumors poses distinct challenges, partly due to the variety of tumors. Correctly identifying skin lesions is crucial in order to avoid missing potential malignancies and to facilitate early treatment, required to minimize morbidity and mortality. Histopathological examination serves as a crucial tool in the diagnosis of skin tumors. Thus, it is essential to correlate clinical characteristics with both gross and microscopic findings.

## Author contributions:

Garima Anandani conceptualized the idea and Payal Bhatt collected the data. Garima Anandani drafted the manuscript and performed the data analysis. Parth Goswami and Yashdeep Singh Pathania contributed in reviewing and supervising the manuscript. All authors contributed to the article and approved the submitted version.

## Ethical statement:

The authors confirm that the ethical policies of the journal have been adhered to. Ethical approval was taken by the Institutional Ethics Committee (IEC Approval number: AIIMS/RAJKOT/5th/ER/12). No identification details of the patient have been shared in the article.

## Funding:

No funds were received or used.
